# Sex-Based Differences in Dissection Patterns and Surgical Strategies in Tear-Oriented Repair for Acute Type A Aortic Dissection: A Single-Center Retrospective Study

**DOI:** 10.5761/atcs.oa.25-00182

**Published:** 2026-01-22

**Authors:** Ryumon Matsumoto, Taiju Watanabe, Ryoji Kinoshita, Kazunobu Hirooka

**Affiliations:** Department of Cardiovascular Surgery, Tsuchiura Kyodo General Hospital, Tsuchiura, Ibaraki, Japan

**Keywords:** acute aortic dissection, sex differences, tear-oriented surgery, tear-oriented surgery

## Abstract

**Purpose:**

This study aimed to evaluate sex-based differences in the clinical characteristics, aortic anatomy, surgical strategies, and outcomes of patients undergoing emergency surgery for acute Stanford type A aortic dissection (AAAD).

**Methods:**

We retrospectively analyzed 148 consecutive patients (82 males and 66 females) who underwent surgery for AAAD at a single center. We compared their backgrounds, entry tear locations, operative procedures, and postoperative outcomes. Kaplan–Meier analysis assessed long-term survival and freedom from re-intervention.

**Results:**

Female patients were significantly older than male patients, more likely to have ascending aortic entry tears, and more often treated by hemiarch replacement. Male patients underwent total arch replacement more frequently because of arch or distal entry tears and had a higher incidence of iliac artery involvement, indicating more extensive distal dissection. In-hospital mortality, major postoperative complications, long-term survival, and freedom from re-intervention showed no significant sex-based differences.

**Conclusion:**

In female patients, the predominance of ascending entry tears allows less extensive surgery without compromising outcomes. Therefore, when dissection patterns are suitable, emergent surgery is appropriate even in elderly female patients.

## Introduction

Acute Stanford type A aortic dissection (AAAD) is a life-threatening condition with high early mortality (approximately 60%) if not treated surgically.^1,2)^

The International Registry of Acute Aortic Dissection shows that surgical outcomes have improved over time, with in-hospital mortality decreasing from 31% to 22% and surgical mortality from 25% to 18% over a 17-year period.^3)^ In Japan, even more favorable outcomes have been reported. Ogino et al.^4)^ reported an in-hospital mortality rate of approximately 10% as of 2023. However, sex was not identified as an independent risk factor in their multivariable model, and sex-specific analyses remain limited in general.

Given these improved outcomes, a re-evaluation of sex-based differences in clinical presentation, anatomical features, and surgical strategy is warranted. Anatomical factors, such as entry tear location, aortic diameter, and dissection extent, have been shown to influence the choice of surgical technique (e.g., hemiarch replacement [HAR] vs. total arch replacement [TAR]) and long-term outcomes. These anatomical variables may differ by sex, but their clinical implications have not been fully elucidated.

We conducted a retrospective study of patients who underwent emergency surgery for AAAD at our institution. Our primary aim was to evaluate the effect of sex on clinical characteristics, anatomical patterns, surgical strategies, and outcomes. In particular, we examined whether tear-oriented surgical approaches were equally effective in both sexes, particularly among elderly female patients, who more frequently present with proximal entry tears and are often considered higher-risk candidates for surgery.

## Materials and Methods

### Study design and population

This single-center retrospective study was conducted at Tsuchiura Kyodo General Hospital. This study included consecutive patients who underwent emergency surgical repair for AAAD between January 2013 and December 2022 and had complete preoperative imaging and intraoperative data. The diagnosis of AAAD was confirmed by contrast-enhanced computed tomography (CT) of the thoracic aorta. Patients were excluded if critical data regarding the location of the primary entry tear or the distal extent of dissection were unavailable. In total, 148 patients (82 males and 66 females) who underwent emergency surgery for AAAD were included.

### Surgical procedures

All surgeries were performed via median sternotomy under general anesthesia. Cardiopulmonary bypass was established using femoral artery perfusion and right atrial drainage, although axillary artery perfusion was used in selected cases. Systemic cooling was initiated and maintained until a bladder temperature of 26°C–28°C was reached. Cardiac arrest was achieved using a combination of antegrade and retrograde cardioplegia, and selective antegrade cerebral perfusion was used for cerebral protection during circulatory arrest.

During the cross-clamping of the ascending aorta, a proximal stump was prepared. Distal anastomosis was performed in an open distal fashion under circulatory arrest. The extent of aortic replacement was determined according to the location of the primary entry tear. When a tear was located in the ascending aorta, the replacement was limited to the ascending aorta. If an entry tear was present in or extended to the aortic arch, partial or TAR was performed. In cases in which no entry tear was detected in the ascending aorta or arch, ascending aortic replacement alone was selected.

### Data collection and variable definitions

Demographic, clinical, and operative data were collected from electronic medical records. The variables included sex, age, body mass index (BMI), comorbidities, primary entry tear location, distal extent of dissection, surgical procedure, and laboratory parameters.

The location of the primary entry tear was determined on the basis of intraoperative findings and preoperative CT imaging and was categorized into the ascending aorta, aortic arch, or descending aorta. The distal extension of the dissection was anatomically classified into the thoracic descending aorta, abdominal aorta, and iliac arteries.

### Statistical analysis

All statistical analyses were performed using R version 4.3.2 (R Foundation for Statistical Computing, Vienna, Austria). Continuous variables were expressed as mean ± standard deviation or median (interquartile range) and were compared using Student’s t-test or the Mann–Whitney U-test depending on the distribution. Categorical variables were presented as frequencies (percentages) and were compared using the chi-squared test or Fisher’s exact test as appropriate.

The primary analysis focused on comparing the anatomical characteristics and perioperative variables between male and female patients. A 2-sided p-value <0.05 was considered statistically significant.

In addition, to evaluate whether female sex was independently associated with in-hospital mortality, a multivariable logistic regression analysis was performed, including age and sex as clinically relevant covariates.

### Ethical considerations

This study was approved by the Ethics Committee of Tsuchiura Kyodo General Hospital (approval no. 2025FY22) and was conducted in accordance with the Declaration of Helsinki. The requirement for written informed consent was waived owing to the retrospective design of the study.

## Results

**Table 1** shows a summary of the baseline characteristics of the patients.

**Table 1:** Comparison of preoperative characteristics between male and female patients undergoing emergency surgery for acute Stanford type A aortic dissection

	No. of patients (%)/mean ± SD		p-Value
	Male (n = 82)	Female (n = 66)	
Age (years)	59.7 ± 12.3	73.6 ± 9.7	<0.001
BMI (kg/m^2^)	25.7 ± 4.0	23.0 ± 4.1	<0.001
Smoking history	48 (58.5)	17 (25.8)	<0.001
Hypertension	33 (40.2)	37 (56.1)	0.069
Diabetes mellitus	5 (6.1)	3 (4.5)	0.732
History of ischemic heart disease	2 (2.4)	1 (1.5)	1
History of cerebral vascular disease	3 (3.7)	12 (18.2)	0.01
Preoperative coma	5 (6.1)	4 (6.1)	1
Limb malperfusion	4 (4.9)	2 (3.0)	0.692
Visceral malperfusion	4 (4.9)	4 (6.1)	1
Cerebral malperfusion	7 (8.5)	6 (9.1)	1
Coronary malperfusion	3 (3.7)	0 (0)	0.254
Shock vital signs	8 (9.8)	7 (10.6)	1
Pericardial effusion	17 (20.7)	27 (40.9)	0.011
Pleural effusion	5 (6.1)	10 (15.2)	0.099
Connective tissue disease	4 (4.9)	1 (1.5)	0.381
Bicuspid aortic valve	1 (1.2)	1 (1.5)	1
Location of primary entry tear			
Ascending aorta	45 (54.9)	48 (72.7)	0.025
Aortic arch	27 (32.9)	12 (18.2)	0.060
Descending aorta	10 (12.2)	6 (9.1)	0.604
Distal extent of dissection			
Ascending aorta	6 (7.3)	7 (10.6)	0.563
Aortic arch	5 (6.1)	11 (16.7)	0.060
Thoracic descending aorta	3 (3.7)	6 (9.1)	0.188
Aorta abdominalis	22 (26.8)	22 (33.3)	0.470
Iliac artery	45 (54.9)	22 (33.3)	0.003
Patent false lumen	77 (93.9)	52 (89.4)	0.012
Surgical procedure			
HAR	52 (63.4)	59 (89.4)	<0.001
PAR	3 (3.7)	3 (4.5)	1
TAR	27 (32.9)	4 (6.1)	<0.001
Concomitant procedures			
CABG	5 (6.1)	1 (1.5)	0.226
Bentall	9 (11.0)	1 (1.5)	0.043
David	2 (2.4)	0 (0)	0.502
Laboratory data			
Creatinine (mg/dL)	1.25 ± 0.93	1.17 ± 1.29	0.672
T-bilirubin (mg/dL)	0.89 ± 0.44	0.83 ± 0.40	0.360
Albumin (g/dL)	3.84 ± 0.50	3.74 ± 0.37	0.185
White blood cell count (/μL)	12576 ± 4273	10520 ± 4368	0.005
Neutrophil count (/μL)	9936 ± 4308	8346 ± 4410	0.032
Lymphocyte count (/μL)	1859 ± 1388	1532 ± 917	0.093
Platelet count (×10^4^/μL)	19.01 ± 6.66	17.04 ± 6.05	0.062

SD: standard deviation; BMI: body mass index; HAR: hemiarch replacement; PAR: partial arch replacement; TAR: total arch replacement; CABG: coronary artery bypass grafting

### Patient characteristics

The female patients participating in this study were significantly older than the male patients (73.6 ± 9.7 vs. 59.7 ± 12.3 years, p <0.001) and had lower BMI values (23.0 ± 4.1 vs. 25.7 ± 4.0 kg/m^2^, p <0.001). Smoking history was more prevalent in male patients (58.5%) than in female patients (25.8%, p <0.001). No significant sex-related differences were observed regarding the prevalence of hypertension or diabetes mellitus, but female patients had a higher incidence of prior cerebrovascular disease than male patients (18.2% vs. 3.7%, p = 0.01). Other preoperative clinical parameters, including coma, malperfusion syndrome, and shock vital signs, did not differ significantly between the groups; however, pericardial effusion occurred more frequently in female patients than in male patients (40.9% vs. 20.7%, p = 0.011).

### Anatomical characteristics of dissection

The location of the primary entry tear differed according to sex. Female patients had a significantly higher proportion of ascending aortic entries (72.7% vs. 54.9%, p = 0.025), whereas male patients tended to have more arch entries (32.9% vs. 18.2%, p = 0.060). Regarding the distal extent of dissection, iliac artery involvement was significantly more frequent in male patients than in female patients (54.9% vs. 33.3%; p = 0.003). The patency rate of the false lumen was also higher in male patients than in female patients (93.9% vs. 89.4%, p = 0.012).

### Surgical procedures

TAR was performed more frequently in male patients than in female patients (32.9% vs. 6.1%, p <0.001), whereas HAR was more common in female patients than in male patients (89.4% vs. 63.4%, p <0.001). The rates of partial arch replacement (PAR) and concomitant procedures (coronary artery bypass grafting, Bentall, and David) did not differ significantly between the 2 groups.

### Laboratory findings

White blood cell (WBC) and neutrophil counts were significantly higher in male patients (WBC: 12576 ± 4273 vs. 10520 ± 4368/μL, p = 0.005; neutrophils: 9936 ± 4308 vs. 8346 ± 4410/μL, p = 0.032). Although albumin and platelet counts showed no significant sex differences, the prognostic nutritional index (PNI) and geriatric nutritional risk index (GNRI) were both significantly higher in male patients (PNI: 47.4 ± 9.2 vs. 44.9 ± 6.3, p = 0.045; GNRI: 105.7 ± 10.8 vs. 99.3 ± 9.1, p <0.001). Inflammatory ratios, such as the neutrophil-to-lymphocyte ratio and platelet-to-lymphocyte ratio, did not differ between sexes.

### Postoperative outcomes

**Table 2** shows a summary of the postoperative outcomes. The incidence of prolonged mechanical ventilation (≥72 hours) was identical between males and females (13.4% vs. 16.7%, p = 0.6455). The rates of stroke (11.0% vs. 13.6%, p = 0.6244), spinal cord injury (4.9% vs. 1.5%, p = 0.3813), dialysis requirement (6.1% vs. 9.1%, p = 0.5406), and pericardial drainage (3.7% vs. 1.5%, p = 0.6288) did not differ significantly between the sexes.

**Table 2:** Sex-based differences in postoperative outcomes and hospital stay

	No. of patients (%)/mean ± SD		p-Value
	Male (n = 82)	Female (n = 66)	
Postoperative ventilation ≥72 hours)	11 (13.4)	11 (16.7)	0.646
Stroke	9 (11.0)	9 (13.6)	0.624
Spinal cord injury	4 (4.9)	1 (1.5)	0.381
Dialysis requirement	5 (6.1)	6 (9.1)	0.541
Pericardial drainage required	3 (3.7)	1 (1.5)	0.629
72-hour mortality	4 (4.9)	3 (4.5)	1
In-hospital mortality	11 (13.4)	5 (7.6)	0.297
ICU stay (days)	4.30 ± 4.32	5.74 ± 9.23	0.251
Hospital stay (days)	28.7 ± 24.1	26.3 ± 18.3	<0.001

SD: standard deviation; ICU: intensive care unit

Early mortality within 72 hours was observed in 4 male patients (4.9%) and 3 female patients (4.5%), with no statistically significant difference (p = 1.000). Similarly, the overall in-hospital mortality rate did not differ significantly between male and female patients (13.4% vs. 7.6%, p = 0.2971).

Although the median length of stay in the intensive care unit was slightly longer in female patients than in male patients (5.7 vs. 4.3 days, p = 0.2509), this difference was not statistically significant. However, male patients had a significantly longer mean length of stay in the hospital than female patients (28.7 vs. 26.3 days, p <0.001).

To assess whether female sex was independently associated with early mortality, a multivariable logistic regression analysis adjusting for age was performed.

Female sex was not an independent predictor of in-hospital mortality (odds ratio 0.59, 95% confidence interval 0.15–2.09, p = 0.43).

### Long-term outcomes

**Fig. 1:**
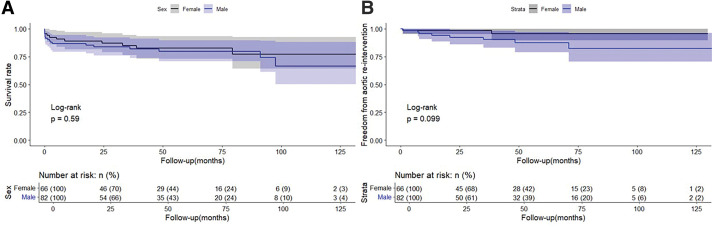
Long-term outcomes by sex. Kaplan–Meier curves showing overall survival (**A**) and freedom from aortic re-intervention (**B**) in male (blue) and female (gray) patients. No significant differences were observed between sexes (log-rank p = 0.59 and 0.099, respectively).

Kaplan–Meier analysis revealed that there was no significant difference in overall survival between male and female patients (log-rank p = 0.59; **Fig. 1A**). Similarly, freedom from aortic re-intervention did not differ significantly by sex, although a non-significant trend toward lower re-intervention-free survival was observed in male patients (log-rank p = 0.099; (**Fig. 1B**).

**Fig. 2:**
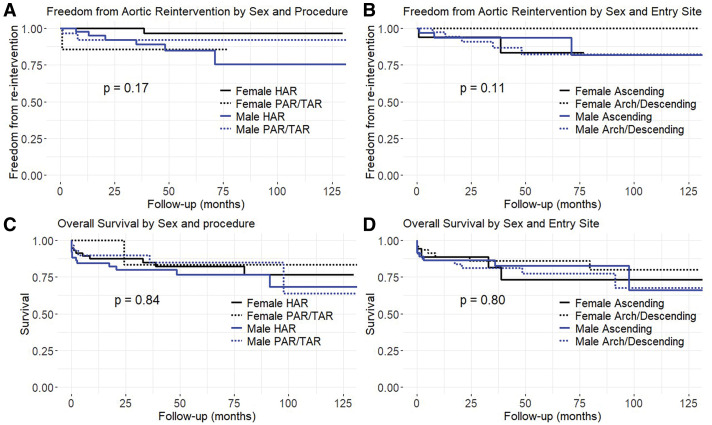
Stratified Kaplan–Meier analysis by sex, procedure, and entry site. Freedom from aortic re-intervention (**A**, **B**) and overall survival (**C**, **D**) stratified by sex and surgical procedure (HAR vs. PAR/TAR; **A**, **C**), and by sex and entry site (ascending vs. arch/descending; **B**, **D**). No significant differences were observed in any subgroup (log-rank p = 0.17, 0.11, 0.84, and 0.80, respectively). HAR: hemiarch replacement; PAR: partial arch replacement; TAR: total arch replacement

To further explore the interaction between sex and surgical factors, we stratified the Kaplan–Meier analyses by surgical procedure (HAR vs. PAR/TAR) and entry tear location (ascending vs. arch/descending) for each sex (**Fig. 2**).

Neither survival nor freedom from re-intervention showed significant differences among the 4 groups in either comparison.

Specifically, no statistically significant differences were observed in re-intervention-free survival when patients were grouped by sex and procedure (p = 0.17; **Fig. 2A**) or by sex and entry site (p = 0.11; **Fig. 2B**).

Similarly, overall survival did not differ significantly by sex and procedure (p = 0.84; **Fig. 2C**) or by sex and entry site (p = 0.80; **Fig. 2D**).

## Discussion

In this study, we investigated the sex-based differences in the clinical characteristics and surgical outcomes of patients who underwent emergency surgery for AAAD. Although the preoperative profiles and dissection patterns differed by sex, the comparable short- and long-term outcomes support the effectiveness of a tear-oriented surgical strategy. The female patients were significantly older at presentation, consistent with previous reports of delayed AAAD onset in female patients.^5–7)^ Despite advanced age, the postoperative complication and mortality rates in female patients were similar to those in male patients, which supported the feasibility of emergent surgery in elderly female patients (**Table 2** and **Figs. 1** and **2**).

To further assess whether sex itself influenced early mortality, we performed a multivariable logistic regression analysis adjusting for age. Female sex was not identified as an independent predictor of in-hospital mortality (odds ratio 0.59, 95% confidence interval 0.15–2.09, p = 0.43), indicating that the observed similarity in early outcomes was not attributable to confounding by age. This finding suggests that anatomical characteristics and the application of a tear-oriented surgical strategy may mitigate potential age-related disadvantages in female patients.

Several factors may contribute to the age-related sex differences in AAAD. Estrogen may preserve vascular compliance and connective tissue integrity, delaying disease progression.^8–10)^ Additionally, younger female patients tend to have smaller aortas but may experience faster aortic growth over time.^11,12)^ Lifestyle differences, such as lower smoking rates, and the comparable prevalence of genetically triggered aortopathy between sexes may also play a role.^13)^

In addition to age, the dissection patterns also differed markedly. Female patients more frequently had primary entry tears located in the ascending aorta and showed limited distal extension, whereas male patients more often had entry sites in the arch or more distal segments and exhibited greater propagation into the iliac arteries. These findings suggest a tendency toward more localized dissections in female patients and more extensive propagation in male patients (**Table 1**). These anatomical trends influenced the surgical strategy: male patients more often underwent total arch or root replacement, whereas female patients more frequently underwent limited repairs, such as HAR. This sex-related difference in surgical procedures was not only observed in our study but has also been noted in previous reports, including a recent meta-analysis.^12,14)^

Several mechanisms may explain this difference. Younger male patients may have more elastic aortas, thus allowing for greater false lumen propagation, whereas older female patients may have stiffer and degenerated aortic walls, thus preventing extensive distal progression of the dissection.^15)^ This finding may also explain the higher incidence of pericardial effusion in female patients (**Table 1**). Biomechanical studies further suggest that aortas in males have thicker media and greater tensile strength, whereas those in females exhibit reduced delamination resistance and wall strength with advancing age.^16)^ Differences in hemodynamics and arch geometry may also affect dissection patterns, although direct evidence is limited.^14)^

These anatomical characteristics are clinically relevant. When the entry tear is in the ascending aorta, which is more common in female patients, HAR may be sufficient and reduce the operative morbidity, even in elderly patients. By contrast, distally located tears in male patients often require more extensive procedures, thus emphasizing the value of individualized, tear-oriented planning.

Surgical approaches for AAAD may vary, with some centers favoring routine TAR and others preferring entry-oriented strategies. Although high-volume centers can safely perform TAR, recent meta-analyses suggest that selective resection based on tear location offers comparable outcomes and fewer complications.^17)^ In the current study, procedures were selected according to entry location, and in-hospital and long-term outcomes did not differ significantly according to the type of procedure or the sex of the patient (**Figs. 1** and **2**).

Notably, despite being older and having more comorbidities, female patients had a significantly shorter postoperative length of stay in the hospital than male patients (**Tables 1** and **2**). Stratified survival analysis showed that there were no differences across sex-specific subgroups, thus reinforcing the consistency of outcomes under entry-oriented surgical strategies (**Fig. 2**).

### Limitations

This study has several limitations. First, this was a single-center retrospective study with a limited sample size, which may reduce the generalizability of the findings. Second, only patients who underwent emergency surgery were included; patients who died before surgery or were deemed unsuitable for operative management were not captured, introducing a potential survival bias. This should be considered when interpreting sex-related differences in clinical presentation and outcomes. Third, follow-up attrition beyond 5 years may have influenced the assessment of late outcomes. Finally, data on frailty and functional status were unavailable, which may also affect the interpretation of sex-based differences.

## Conclusion

Tailoring surgery to the location of the primary intimal tear yields favorable outcomes regardless of sex. Tear-oriented repair appears to be particularly well suited for female patients with ascending tears, thus enabling less invasive procedures without compromising durability. In male patients, a more extensive disease often necessitates broader surgical intervention; however, the outcomes remain comparable. These findings support tear-oriented surgery as a sound and individualized strategy for both sexes. Future research should validate our findings via multicenter prospective studies.

## Declarations

### Ethics approval and consent to participate

This study was approved by the Ethics Committee of Tsuchiura Kyodo General Hospital (approval no. 2025FY22), and the requirement for written informed consent was waived owing to the retrospective design of the study.

### Consent for publication

Not applicable.

### Funding

This research received no external funding.

### Conflicts of interest

The authors declare no conflicts of interest.

### Data availability

The data that support the findings of this study are available from the corresponding author upon reasonable request.

### Authors’ contributions

RM designed the study and wrote the manuscript. TW, RK, and KH contributed to data collection and analysis.

All authors have read and approved the final version of the manuscript.
